# The alternative complement pathway aids in vascular regression during the early stages of a murine model of proliferative retinopathy

**DOI:** 10.1096/fj.15-280834

**Published:** 2015-11-30

**Authors:** Clifford Kim, Kaylee E. Smith, Alexandra Castillejos, Daniel Diaz-Aguilar, Magali Saint-Geniez, Kip M. Connor

**Affiliations:** *Angiogenesis Laboratory, Massachusetts Eye and Ear Infirmary, Boston, Massachusetts, USA; ^†^Department of Ophthalmology, Harvard Medical School, Boston, Massachusetts, USA; and ^‡^Schepens Eye Research Institute, Boston, Massachusetts, USA

**Keywords:** Cd55, Cd59, factor b, innate immune system, OIR, Vegf

## Abstract

Proliferative retinopathic diseases often progress in 2 phases: initial regression of retinal vasculature (phase 1) followed by subsequent neovascularization (NV) (phase 2). The immune system has been shown to aid in vascular pruning in such retinopathies; however, little is known about the role of the alternative complement pathway in the initial vascular regression phase. Using a mouse model of oxygen-induced retinopathy (OIR), we observed that alternative complement pathway–deficient mice (*Fb^−/−^*) exhibited a mild decrease in vascular loss at postnatal day (P)8 compared with age- and strain-matched controls (*P* = 0.035). Laser capture microdissection was used to isolate the retinal blood vessels. Expression of the complement inhibitors *Cd55* and *Cd59* was significantly decreased in blood vessels isolated from hyperoxic retinas compared with those from normoxic control mice. *Vegf* expression was measured at P8 and found to be significantly lower in OIR mice than in normoxic control mice (*P* = 0.0048). Further examination of specific *Vegf* isoform expression revealed a significant decrease in *Vegf120* (*P* = 0.00032) and *Vegf188* (*P* = 0.0092). In conjunction with the major modulating effects of Vegf during early retinal vascular development, our data suggest a modest involvement of the alternative complement pathway in targeting vessels for regression in the initial vaso-obliteration stage of OIR.—Kim, C., Smith, K. E., Castillejos, A., Diaz-Aguilar, D., Saint-Geniez, M., Connor, K. M. The alternative complement pathway aids in vascular regression during the early stages of a murine model of proliferative retinopathy.

The formation of new blood vessels from pre-existing vessels, called angiogenesis, is a highly regulated process that depends on the appropriate interaction of a variety of proteins, such as Vegf and hypoxia-inducible factor (HIF)-1α (1, 2). Any imbalances in these proteins can cause abnormal angiogenesis, which could lead to permanent ocular damage. Abnormal angiogenesis is a hallmark of many ocular diseases, such as diabetic retinopathy and retinopathy of prematurity (ROP) (3, 4). The murine model of oxygen-induced retinopathy (OIR) is a biphasic model similar to ROP, which is associated with late-stage destructive neovascularization (NV) and inflammation (3, 5). The first phase of OIR is precipitated by relative hyperoxia (3), resulting in cessation of normal retinal vessel growth and dropout of pre-existing vasculature, leaving a centrally avascular retina (3, 5, 6). This effect is driven primarily by the suppression of oxygen-sensitive growth factors during this phase of disease (3, 5). The initial loss of retinal vasculature, coupled with the increasing metabolic demands of the developing retina, leads to local hypoxia and the up-regulation of angiogenic growth factors. As a consequence, there is an overcompensating vasoproliferative response with the formation of disorganized and leaky neovascular tufts, the hallmark of phase 2. It is this pathologic NV and subsequent complications (*e.g.,* bleeding and retinal distortion) that can lead to visual impairment. Although in most cases of ROP the neovascular tufts regress after the underlying retina revascularizes, in some patients the pathologic angiogenic insult is too severe, resulting in a retinal detachment. After detachment, individuals often remain severely visually impaired, making ROP one of the leading causes of pediatric blindness (7, 8). Although the incidence of ROP is ∼2000 infants per year, ROP continues to become more prevalent, especially in developing countries, most likely because of advances in medical technology that enable more premature infants to survive (7). Thus, ROP is a disease that warrants further investigation.

As in ROP, OIR leads to a similar hypoxic environment that triggers the development of neovascular tufts (**Fig. 1**) (5, 6). A major distinction between human ROP and the mouse OIR model is the initial avascular event. When exposed to high oxygen, mouse retinas exhibit extensive vaso-obliteration (VO), whereas human retinas predominantly undergo vascular growth cessation with minimal VO. However, OIR mirrors phase 2 of human ROP, with the formation of neovascular tufts. Recent studies of this model indicate that NV coincides with increased activity of the innate immune system (9, 10). The complement system facilitates the removal of damaged tissue and nonhost cells and consists of 3 pathways: classical, lectin, and alternative (11). The classical pathway is mediated by the binding of the complement component (C)1q protein complex to antigen–antibody complexes, whereas the lectin pathway is regulated by mannose-binding lectin’s (Mbl) recognition of polysaccharide or glycoprotein motifs on the cell surface of nonhost cells (12). Last, the alternative complement pathway is constitutively active through the spontaneous cleavage of the C3 thioester bond into C3a and C3b subunits (13). Further continuation of the alternative complement pathway is allowed only by complement factor b (Fb) deposition, which targets cells for removal by the binding of its active form Bb to C3b (12–14). These components form the C3 convertase enzyme, promoting the cleavage of C3 and creating a positive feedback loop (13) that can be down-regulated by complement inhibitors such as cluster of differentiation (Cd)55 (15) and Cd59 (16, 17). The next step of the pathway is the production of the C5 convertase, created by the combination of the C3 convertase with an additional C3b molecule. The C5 convertase then cleaves C5 into C5a and C5b (12, 13). C5b localizes to the cell surface where it recruits C6, -7, -8, and -9, creating the membrane attack complex (MAC), which forms a pore in the cell membrane causing cell lysis and death, thus facilitating tissue removal (12).

**Figure 1.**
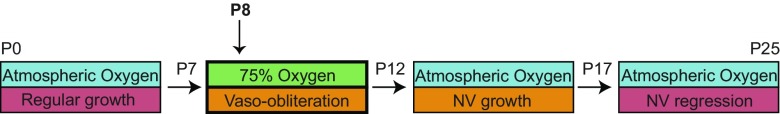
Mouse model of OIR. Timeline depicts mouse postnatal age (P) in days and amount of time spent either in atmospheric room air (blue) or 75% O_2_ (green), to induce angiogenesis. Mouse retinas were analyzed at P8. The corresponding vascular changes that take place through the course of disease are labeled below the time course. Pink: normal vascular changes; orange: neovascular changes.

Research from our laboratory has uncovered an important role of the alternative complement system in the targeted removal and resolution of pathologic NV in phase 2 of the OIR model (18). In this study, alternative pathway-deficient mice (*Fb*^−/−^) exhibited an increase in neovessel development, as well as an increase in regression time of pathologic NV compared to wild-type (WT) controls, which indicated some level of involvement of the alternative pathway in the development and regression of the phase 2 neovascular tufts. Of particular interest, WT mice undergoing OIR showed decreased expression of the complement inhibitor *Cd55* within the neovascular tufts compared to normoxic controls (18). Taken together, these findings strongly suggest that the alternative complement pathway specifically targets neovascular tufts for removal. This work led us to question whether a similar mechanism is involved in the initial VO phase of this disease. It has been established that the severity of VO in phase 1 is indicative of the severity of pathologic NV in phase 2 (3). Because of this relationship, the alternative complement pathway may also have a role earlier in the pathologic course of disease. Thus, we investigated the potential effect of the alternative complement pathway in the ocular pathogenesis of vessel regression during the initial (vascular dropout) phase of OIR (7). Examining this relationship between the complement pathway and vascular dropout will enhance our understanding of ROP and aid in the identification of potential therapeutic targets.

## MATERIALS AND METHODS

### Animals and OIR model

In this study, we followed the guidelines set by the Association for Research in Vision and Ophthalmology (ARVO) Statement for the Use of Animals in Ophthalmic and Vision Research. The C57Bl/6 (stock no. 000664) and *Mbl A/C^−/−^* (stock no. 006122) mice were from Jackson Laboratories (Bar Harbor ME, USA), and the *Fb^−/−^* and *C1q^−/−^* mice were a gift from Dr. J. Lambris (University of Pennsylvania, Philadelphia, PA, USA). Breeding colonies were maintained at the Massachusetts Eye and Ear Infirmary (MEEI) animal facility. We used the murine OIR model, a well-established model of ROP (Fig. 1) (5, 6). Newborn mice and mothers were kept in room air (21% oxygen) from postnatal day (P)0 to P7. At P7 they were placed in a hyperoxic chamber at 75% oxygen (model 110: Biospherix, Lacona, NY, USA) for 24 h, until P8. At this point in the VO stage, the mice were taken out of the oxygen chamber and weighed. Mice under 3 g at P8 were excluded from analysis, to minimize the potential for abnormal phenotypes occasionally seen with inadequately nursed and low-weight mice (5, 18). Mouse pup weight was comparable in all the experimental strains and to that of the littermate controls. Mice were anesthetized with Avertin (T4, 840-2; 2,2,2 tribromoethanol; Sigma-Aldrich, St. Louis, MO, USA, in isoamyl alcohol, A730-1; ThermoScientific, Waltham, MA, USA), at a concentration of 10 mg/ml and injected at a dose of 0.25–0.5 mg/g i.p. The eyes were then enucleated as has been described (5).

### Retinal flatmounts and analysis

After enucleation, the eyes were immediately placed in 4% paraformaldehyde for 1 h at room temperature and then stored in PBS at 4°C. The retinas were carefully dissected as described elsewhere (5), and the hyaloid vasculature was removed. One percent Triton X-100 (T-8787; Sigma-Aldrich) in PBS was used to permeabilize the tissue overnight at 4°C. The tissue was incubated in isolectin B4 conjugated to Alexa Fluor 568 (GS-IB4; 121412; ThermoScientific-Life Technologies, Carlsbad, CA, USA) diluted 1:50 in 1 mM CaCl_2_ solution at 25°C and protected from light overnight on a vacillating platform. After 24 h, the retinas were washed 3 times for 45 min in 1× PBS. They were flat mounted by cutting into a petal shape with 4 lobes and coverslipped with glycerol/PBS (S2828; ThermoScientific-Invitrogen). An AxioVision microscope (Zeiss, Thornwood, NY, USA) was used to take tiled images of the retinal flatmount at ×5 magnification. The tiles were stitched together by AxioVision 4.8.2 software. The stitched images were saved as TIFF files and opened in PhotoShop (version CS5; Adobe Systems, San Jose, CA, USA) (5) for quantification. By encircling the entire vascular flatmount area and the VO areas separately with the Quick Selection tool, we calculated the percentage of VO by dividing the number of pixels in the vaso-obliterated area from the number of pixels in the total retinal flatmount vascular area and multiplied by 100.

### Laser capture microdissection

Eyes were enucleated and embedded in optimal cutting temperature compound (Tissue Tek; Sakura Finetek, Torrance, CA, USA), frozen in cold isopropanol on dry ice, and stored at −80°C. They were cryosectioned at 30 µm into 16 to 18 sections and placed on frame slides (11505190; Leica Microsystems, Wetzlar, Germany). The sections were fixed in an ethanol (459836-1L; Sigma-Aldrich) gradient. Slides were first submerged in 50% ethanol for 1 min, 75% for 1 min, and water for 1 min at 25°C. For staining, the vessel sections were placed in a 1:50 dilution of isolectin B4 HRP (L5391; Sigma-Aldrich) in 1 mM CaCl_2_ for 15 min at 25°C. The slides were then washed in PBS and then 3,3'-diaminobenzidine (DAB; K3467; DakoCytomation-Agilent, Carpinteria, CA, USA) was applied for 2 min. Cell layers were stained by the addition of 0.1% toluidine blue solution (89640-5G; Fluka, St. Louis, MO, USA) for 20 s. The sections were submerged in water 2 times for 30 s each, 75% ethanol for 30 s, and 100% ethanol for 30 s. Sections were dried in room air for 5 min. Laser capture microdissection (LCM; LMD 7000; Leica Microsystems) was used to isolate retinal vasculature and the ganglion cell layer (GCL). Samples were collected in 50 µl RNAlater (AM7022; Thermo Scientific-Ambion), followed by RNA isolation and RT-PCR.

### RT-PCR for LCM

RNA was isolated from the retinal vasculature and the GCL and collected by LCM. RNA isolation was performed with the RNeasy micro kit (74004; Qiagen, Valencia, CA, USA). Samples underwent cDNA synthesis by Superscript III (18080-044; Thermo Scientific-Invitrogen). RT-PCR was performed with KAPA SYBR Fast Universal Master Mix (KK4600; KAPA Biosystems, Wilmington, MA, USA) on a Step One Plus real-time PCR system (ThermoScientific-Applied Biosystems). Primers for *Cd55* and *Cd59* were created by the online Primer Quest design tool and bought from Integrated DNA Technologies (Coralville, IA, USA). Triplicate wells for each sample were run for RT-PCR, and the average threshold cycle (*C_T_*) value was used for analysis. All *C*_T_ values were normalized to the internal control of β-actin. Final analysis was performed by the ^∆∆^*C_T_* method.

### RT-PCR for whole retina

The retina was isolated and flash frozen in liquid nitrogen. The RNeasy mini kit (74106; Qiagen) was used to isolate RNA from the whole retina. A Nanodrop spectrometer (ThermoScientific) was used to determine the RNA concentration of the samples. Five hundred nanograms of RNA was synthesized into cDNA by Superscript III (18080-044; ThermoScientific-Invitrogen). Absolute quantification RT-PCR for whole retina was performed with KAPA SYBR Fast Universal Master Mix (KK4600; KAPA Biosystems) on a Step One Plus real-time PCR system (ThermoScientific-Applied Biosystems).

### RT-PCR for *Vegf* isoforms

RT-PCR of *Vegf* isoforms was conducted as previously described (18). mRNA (1 µg) was transcribed with the Iscript kit (Bio-Rad, Hercules, CA, USA). Real-time PCR was performed with FastStart SYBR Green master mix on a LightCycler 480 real-time PCR system (Roche Applied Science, Indianapolis, IN, USA). Expression of *Vegf* isoforms was determined by absolute quantification after normalization to housekeeping genes (*B2m*, *Hprt1*, and *Ppia)*. A standard curve, derived from the serial dilution (3 × 10^2^ to 3 × 10^7^ copies/reaction) of a linearized plasmid coding for each isoform (*Vegf188*, *-164*, and *-120*) was created for each reaction and amplified by SYBR Green system, as described earlier. The level of isoform expression in each retina was calculated relative to the standard curve. Results were recorded as means ± sd.

### Statistical analysis

An unpaired Student’s *t* test was used to analyze the data, and results are expressed as means ± sd. Significance was noted as *P* < 0.05.

## RESULTS

### Alternative pathway–deficient mice exhibit decreased vascular loss in phase 1 of OIR

**Figure 2.**
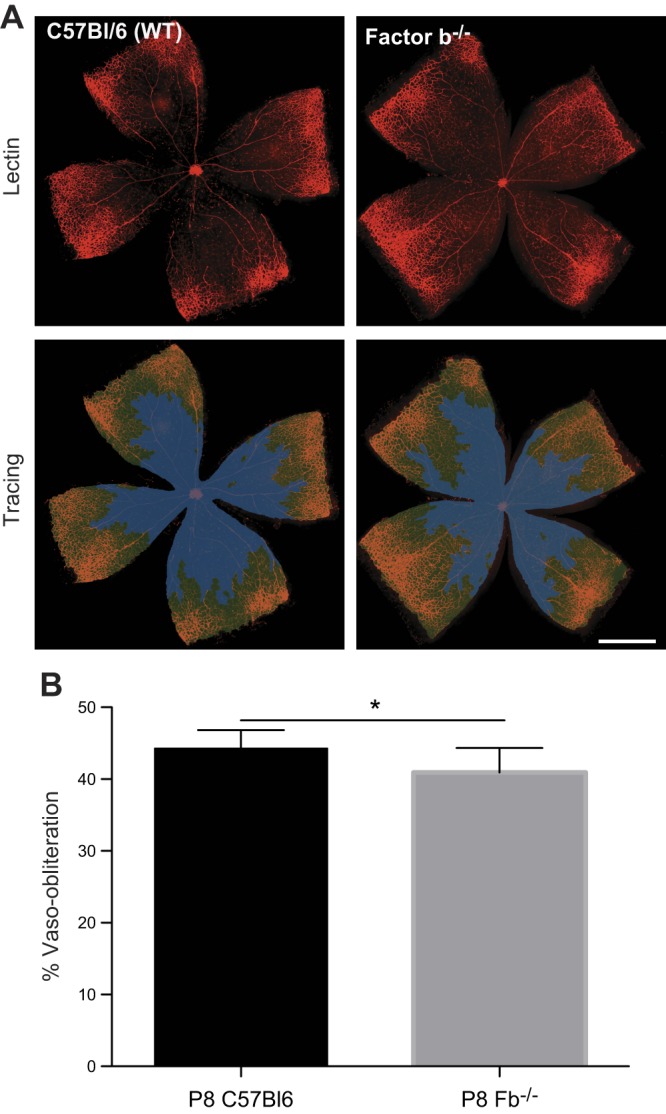
Vascular imaging and quantification of P8 VO in OIR. *A*) Top: representative images of P8 *Fb^−/−^* and WT retinal flatmounts stained for endothelial cells with isolectin B_4_-568. Bottom: tracings of the retina and vaso-obliterated areas. *B*) Quantification of percent VO in *Fb^−/−^* mice compared to WT control mice (C57Bl/6) in OIR at P8 measured as the total area of VO relative to the total retinal vascular area in the flatmount after vascular labeling by isolectin. WT control mice had a statistically significant increase in percentage of VO *vs.* *Fb^−/−^* mice. WT (*n =* 8); *Fb*^−/−^ (*n =* 10). Error bars, SD; *P < 0.05. Scale bar, 1 mm.

To elucidate the role of the alternative complement pathway in retinal VO caused by hyperoxia, we used the mouse model of OIR and mice deficient in the alternative complement pathway (*Fb^−/−^*, **Fig. 2**) (5, 6). Because studies have shown that inhibition of phase 1 can dampen or even prevent progression to sight-threatening phase 2 of ROP (19), this study primarily focuses on the initial VO stage of OIR. At P8, mice experience phase 1 VO, when high oxygen levels suppress *HIF1α* and proangiogenic factors, such as *Vegf* and *Epo* (1, 2). To uncover the role of the alternative complement pathway, we looked at *Fb^−/−^* and WT mice at P8 during the early VO stage of the mouse model of OIR. We then stained the retinas with isolectin B4-568, a marker of endothelial cells (Fig 2*A*). Upon analysis, *Fb^−/−^* mice had a modest but statistically significant decrease in VO compared to WT controls. [WT mice (*n =* 8) had an average of 44.28 ± 2.54% VO, and P8 *Fb^−/−^* mice (*n =* 10) had 40.93 ± 3.42% VO; *P* = 0.035] (Fig. 2*B*). It is worth noting that *Fb^−/−^* mice reportedly have deficiencies in the classical pathway in addition to the alternative pathway. Because the *C2* and *Fb* genes are adjacent on chromosome 17, it was possible that *C2* had been knocked out unintentionally (20). For this reason, classical knockout mice, *C1q^−/−^,* were also evaluated for VO changes. No difference was noted between the *C1q^−/−^* mice (*n =* 7) and WT controls (*P* = 0.26) (Supplemental Fig. 1). In addition, we explored VO changes for the final complement pathway in lectin pathway knockout mice (*Mbl A/C^−/−^*) and found no difference in percentage of VO between *Mbl A/C^−/−^* mice (*n =* 7) and WT controls (*P* = 0.20) (Supplemental Fig. 2). In light of the aforementioned work, which implicated the alternative pathway in facilitating the clearance of NV during phase 2 of OIR (18), we also examined the potential role of the classical and lectin pathways in NV development and clearance in the same *C1q^−/−^* and *Mbl A/C^−/−^* mouse strains as described above. Assessment of these mice at P17, when NV is at its maximum in the OIR model (5), revealed no significant differences in NV in *C1q^−/−^* (*P* = 0.83; *n =* 20) or *Mbl A/C^−/−^* mice (*P* = 0.71; *n =* 13) compared to WT control mice (*n =* 48) (Supplemental Fig. 3).

### Expression of complement inhibitors *Cd55* and *Cd59* is down-regulated in hyperoxic retinal vessels

**Figure 3.**
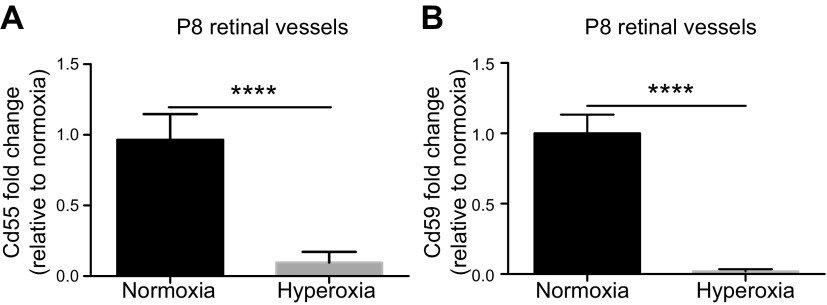
*Cd55* and *Cd59* gene expression in P8 retinal vessels. *Cd55* (*A*) and *Cd59* (*B*) gene expression, found by RT-PCR, in vessels of mice kept in room air (normoxia vessels) as compared to vessels of OIR mice (hyperoxic vessels in regression) at P8 (*n =* 3 eyes/group). Error bars, SD; ****P < 0.0001.

The alternative complement pathway is constitutively active through the presence of the molecule C3, which spontaneously cleaves C3a and -b (13). C3b becomes part of the alternative C3 convertase, which can cleave additional C3 molecules, creating a positive feedback loop in the absence of complement inhibitors (13). Generally, nonhost cells do not produce complement inhibitors, allowing the alternative complement pathway to target these cells for removal. Normal healthy host cells endogenously express complement inhibitors to prevent recognition and targeting by the complement system, thereby evading unnecessary complement-mediated cell death. Cd55 (decay-accelerating factor) and Cd59 are among these critical regulators of the alternative complement pathway. Cd55 inhibits the C3 convertase (15), whereas Cd59 inhibits formation of the MAC (16, 17). To glean whether hyperoxia suppresses complement inhibitors during VO at P8, isolectin-B_4_-stained vessels in WT mice were isolated from retinal sections by LCM. The expression of *Cd55* and *Cd59* in the vessels was determined by RT-PCR (**Fig. 3**). Both *Cd55* (*P* = 4.6 × 10^−10^) and *Cd59* (*P* = 6.0 × 10^−9^) were found to be significantly down-regulated in hyperoxic vessels as compared to normoxic controls. The data suggest that complement has the ability to target these vessels and likely causes increased cell death. This finding is consistent with our aforementioned findings of decreased vascular regression in alternative complement-deficient mice during phase 1 of OIR, strengthening the hypothesis that the alternative complement pathway, in part, mediates vascular dropout (21).

### *Vegf* expression is down-regulated in the hyperoxic retina

**Figure 4.**
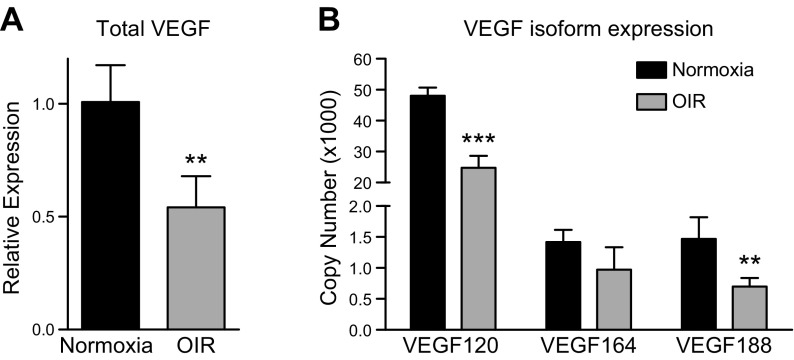
*Vegf* isoform expression in P8 OIR mice. *A*) Total *Vegf* gene expression in whole retinas of mice kept in room air (normoxia) *vs.* OIR mice (hyperoxia) at P8. *B*) *Vegf* isoform expression in whole retinas of mice kept in room air (normoxia) *vs.* OIR mice (hyperoxia) at P8 (*n =* 3-4 eyes/group). Error bars, SD; **P < 0.01; ***P < 0.001.

Vegf is a known, potent driver of angiogenesis in a multitude of diseases, including a variety of retinal pathologies such as age-related macular degeneration, diabetic retinopathy, and ROP. Vascular growth cessation during phase 1 of OIR has been attributed to a stark drop in *Vegf* expression (18, 22). Because of the known importance of Vegf in these ocular diseases, it was necessary to evaluate the expression of *Vegf* in C57Bl/6 P8 OIR mice. As expected, there was a significant decrease in total *Vegf* expression, corresponding to vascular dropout at the P8 time point (*P* = 0.0048) (**Fig. 4*A***). Of interest, expression of *Vegf* isoforms in phase 1 of OIR had not been assessed to date. Because *Vegf* has several different isoforms important in neurovascular development (23), we investigated the expression of 3 isoforms that are prominent in the mouse retina: *Vegf120*, *Vegf164*, and *Vegf188* (18) (Fig. 4*B*). Compared to room air controls, *Vegf120* and *Vegf188* expression declined significantly (*P* = 0.00032 and *P* = 0.0092, respectively) and a downward trend was seen in *Vegf164* (*P* = 0.12). These results strengthen the evidence that Vegf activity plays a major role in early OIR pathogenesis.

## DISCUSSION

Given the consequential nature of pathologic NV after initial VO in the context of OIR, finding a means to suppress or block VO could lessen or inhibit pathologic NV later in disease, thereby reducing the risk of visual impairment. Previous studies have provided evidence that the alternative complement pathway targets neovessels for removal, facilitating vessel regression and disease resolution (18). However, little is known as to whether the alternative complement pathway also plays a role in initial vessel dropout leading to VO.

Retinal flatmounts of WT mice and *Fb^−/−^* mice at P8 were examined to quantify percentage of VO. Less VO occurred in *Fb^−/−^* compared to that in WT controls. Furthermore, expression studies of the complement inhibitors *Cd55* and *Cd59* revealed that inhibitors are suppressed in hyperoxia as compared to normoxia in regressing retinal vessels in mice with OIR at P8. Together, the data suggest that the alternative complement pathway is up-regulated during VO and contributes to vessel dropout. Endothelial cells are known producers of complement, and it is entirely possible that the vasculature stops producing complement inhibitors under these conditions (21). This phenomenon is seen in the rejection of tissue transplants, where the vessels of transplanted organs down-regulate complement inhibitors, allowing them to be targeted by complement and ultimately leading to tissue rejection (24).

The complement system is believed to influence pathologic retinal angiogenesis (25). Specifically, the alternative complement pathway is known to help remove abnormal NV during phase 2 of OIR through the deposition of Fb to neovessels lacking complement inhibitors (18). Our current study suggests that the alternative complement system plays a minor role in disease progression during the first phase of OIR, likely by increased targeting of immature vasculature for VO in response to elevated oxygen, potentially through the same mechanism that enables removal of pathologic neovessels during hypoxia. Although other angiogenic mechanisms are probably at play, given the modest degree of change in VO between mouse strains, alternative complement knockout mice do show some degree of protection. In light of previous data suggesting that alternative complement targets neovascular tufts during late phase OIR, as evidenced by the colocalization of Fb to these tufts in conjunction with down-regulation of complement inhibitors (18), we propose that the clearance of developing vasculature exposed to high oxygen levels during phase 1 occurs *via* the same mechanism: a decrease in complement inhibitors allows for increased Fb to target vessels for removal. Although phases 1 and 2 of OIR occur through opposite mechanisms, both processes show oxygen-dependent changes in complement inhibitor regulation. Thus, it would be interesting to elucidate the mechanism of this oxygen-dependent regulation of complement factors in future studies.

Our study further emphasizes the importance of timing in treating ROP. It is already well established that the degree of VO in phase 1 affects the severity of NV in phase 2 of ROP; increased avascular area manifests into greater NV (3). The converse also holds true; infants with a smaller avascular area develop less NV, allowing normal vessel regression (3). Thus, development of a therapeutic treatment could be beneficial at either phase of ROP, potentially even more so during phase 1. Therefore, therapies targeting the complement system in the setting of retinopathy should attempt to suppress the alternative pathway during the VO phase, while increasing alternative complement activity during NV and vascular repair. However, it is important to note that the alternative complement system is not the only factor involved in VO, as can be seen by the relatively modest percentage of change in VO in *Fb^−/−^* *vs.* that in WT controls at P8. During this early phase of retinal development, the new retinal vessels are still heavily dependent on angiogenic growth factors, such as Vegf. In the hyperoxic conditions of OIR, *HIF1a* is down-regulated, which in turn down-regulates *Vegf* (26, 27). As reported (22) and seen during our evaluation of *Vegf* levels, expression decreases significantly in hyperoxic mice experiencing VO. These results are consistent with findings of a down-regulation of *Vegf* during phase 1, further supporting the notion that VO is heavily driven by the angiogenic factor Vegf (18). It is this drop in *Vegf* expression that most likely accounts for most of the VO and may be why the alternative complement pathway plays only a minor role in facilitating the loss of vessels during phase 1 of OIR. Furthermore, it has been reported that there is no significant difference in retinal *Vegf* expression between *Fb^−/−^* and WT mice during phase 2 of OIR (18), when Vegf is markedly elevated. Given the significant decrease in *Vegf* expression during the early phase of OIR, any potential complemented-related differences in *Vegf* expression are unlikely to contribute to VO. Ultimately, a therapeutic approach that combines alternative complement inhibition and maintenance of angiogenic factors very early in the disease course could ideally prevent the pathologic changes seen in late-stage retinopathy.

## Supplementary Material

Supplemental Data

## Abbreviations

C: complement component
Cd: cluster of differentiation
Fb: complement factor b
GCL: ganglion cell layer
HIF: hypoxia-inducible factor
LCM: laser capture microdissection
MAC: membrane attack complex
Mbl: mannose-binding lectin
NV: neovascularization
OIR: oxygen-induced retinopathy
P: postnatal day
ROP: retinopathy of prematurity
VO: vaso-obliteration
WT: wild-type
