# Supplementary material for: The alternative complement pathway aids in vascular regression during the early stages of a murine model of proliferative retinopathy
Source: FASEB J. 2015 Nov 30;30(3):1300–5. doi: 10.1096/fj.15-280834 (PMC4750413; doi:10.1096/fj.15-280834)
Supplement: Supplemental Data [file supp_30_3_1300__index.html]

The alternative complement pathway aids in vascular regression during the early stages of a murine model of proliferative retinopathy — The alternative complement pathway aids in vascular regression during the early stages of a murine model of proliferative retinopathy — The alternative complement pathway aids in vascular regression during the early stages of a murine model of proliferative retinopathy — Supplemental Data 

# The alternative complement pathway aids in vascular regression during the early stages of a murine model of proliferative retinopathy

## Supplemental Data

- Supplemental Data
- Supplemental Data
- Supplemental Data
