# Supplementary material for: The alternative complement pathway aids in vascular regression during the early stages of a murine model of proliferative retinopathy
Source: FASEB J. 2015 Nov 30;30(3):1300–5. doi: 10.1096/fj.15-280834 (PMC4750413; doi:10.1096/fj.15-280834)
Supplement: Supplemental Data [file supp_fj.15-280834_Supplemental_Figure2.pdf]

Supplemental Figure 2

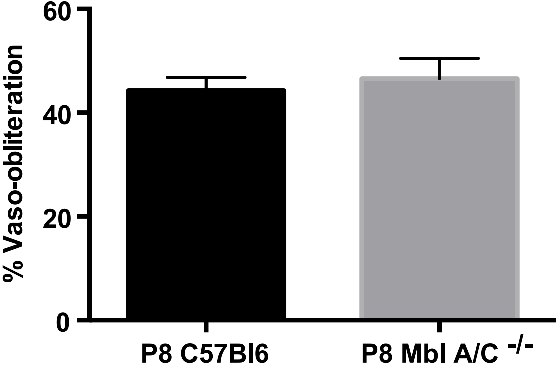

Figure S2. Quantification of OIR P8 VO in *Mbl A/C*$^{-/-}$ mice compared to C57Bl/6 control mice. Quantification of percent vaso-obliteration during phase 1 of OIR at P8 in *Mbl A/C*$^{-/-}$ and C57Bl/6 control mice measured as the total area of VO relative to the total retinal vascular area in the flatmount after vascular labeling by isolectin. There was no statistically significant difference in %VO between the two groups (WT n=8, *Mbl A/C*$^{-/-}$ n=7, P=0.20). Error bars = SD.
