# Supplementary material for: The alternative complement pathway aids in vascular regression during the early stages of a murine model of proliferative retinopathy
Source: FASEB J. 2015 Nov 30;30(3):1300–5. doi: 10.1096/fj.15-280834 (PMC4750413; doi:10.1096/fj.15-280834)
Supplement: Supplemental Data [file supp_fj.15-280834_Supplemental_Figure3.pdf]

Supplemental Figure 3

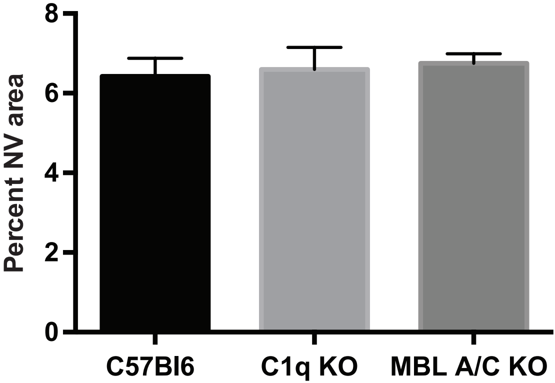

Figure S3. Quantification of OIR P17 NV in *C1q*$^{-/-}$ and *Mbl A/C*$^{-/-}$ mice compared to C57Bl/6 control mice. Quantification of percent neovascular area during phase 2 of OIR at P17 in *C1q*$^{-/-}$, *Mbl A/C*$^{-/-}$, and C57Bl/6 mice measured as the total area of NV relative to the total retinal area in the flatmount after vascular labeling by isolectin. There were no statistically significant differences in %NV area in *C1q*$^{-/-}$ (P=0.83, n=20) or *Mbl A/C*$^{-/-}$ mice (P=0.71, n=13) compared to C57Bl/6 mice (n=48). Error bars = SD.
